# Lessons from the field: Implementing an electronic clinical decision support app for acute febrile illness in rural Cambodia

**DOI:** 10.1093/trstmh/trag035

**Published:** 2026-07-07

**Authors:** Abhijit Mishra, Elke Wynberg, Marco Liverani, Moul Vanna, Phal Chanpheakdey, Chea Nguon, James J. Callery, Bipin Adhikari, Rupam Tripura, Arjun Chandna, Greg Fegan, Naomi Waithira, Thomas J. Peto, Bunreth Voeurng, Chan Davoeng, Huy Rekol, Lek Dysoley, Nicholas P.J. Day, Yoel Lubell, Rusheng Chew

**Affiliations:** a Mahidol Oxford Tropical Medicine Research Unit, Faculty of Tropical Medicine, Mahidol University, Bangkok, 10400, Thailand; b London School of Hygiene & Tropical Medicine, Department of Global Health and Development, London, WC1E 7HT, UK; c National Center for Parasitology, Entomology and Malaria Control, Phnom Penh, 12101, Cambodia; d Centre for Tropical Medicine and Global Health, University of Oxford, Oxford, OX3 7LF, UK; e Cambodia Oxford Medical Research Unit, Angkor Hospital for Children, Siem Reap, 17000, Cambodia; f Action for Health Development, Battambang, 021404, Cambodia; g Faculty of Medicine, University of Queensland, Brisbane, 4072 Australia; h Provincial Health Department, Battambang, 021410, Cambodia

## Abstract

To Improve the management of acute febrile illness in rural low- and middle-income country (LMIC) primary care settings, we developed the Electronic clinical Decision support for Acute fever Management (EDAM) app and evaluated it through a cluster-randomised trial in rural Cambodian primary health centres (PHCs). In parallel, we conducted structured, mixed-methods observations at a sub-set of participating PHCs to document key challenges in screening, enrolling, and managing enrolled patients. A key lesson was the critical importance of user-centred design to align digital tools with health worker workflows and capabilities. The lessons from these observations may help guide researchers and policy-makers developing novel digital health solutions for low- and semi-skilled health workers in rural LMIC settings. Our experiences could offer valuable insights, as there are few documented digital health interventions designed for these contexts. Furthermore, navigating the challenges of implementation and subsequent evaluation remains a significant hurdle for the field.

## Introduction

Acute febrile illness (AFI) is a common presentation in primary health centres (PHCs) across South and Southeast Asia (1). Historically, malaria was the predominant cause of AFI, but its burden has significantly declined, particularly in Cambodia, where malaria cases decreased by 76% between 2015 and 2022 (2). Consequently, the relative burden of non-malarial AFIs has increased considerably (2). This trend exposes significant challenges in their management, at both the individual patient and health system levels.

This is amply illustrated in rural Cambodia, where PHCs are typically staffed by healthcare workers (HCWs) who face significant challenges in diagnosing and treating non-malarial AFI due to limited access to diagnostic tools, reliance on non-user-friendly paper-based guidelines, and limited clinical skill development (3). These factors contribute to the overprescription of antibiotics, a key driver of antimicrobial resistance (AMR), which in 2019, was estimated to directly cause over 3,000 deaths in Cambodia (4).

To help address these problems, we co-created the Electronic clinical Decision support for Acute fever Management (EDAM) app for use in rural PHCs in Battambang province with local medically trained expert clinicians and Provincial Health Department (PHD) officials. The EDAM app is a rule-based algorithm designed to optimise the diagnosis and management of AFI by integrating clinical symptoms, vital signs (including peripheral oxygen saturation), and malaria and C-reactive protein (CRP) rapid test results to formulate a syndromic diagnosis and recommend a management strategy. We aimed to identify the key challenges and lessons learned from implementing a pragmatic cluster-randomised trial of EDAM in 30 PHCs, with the primary outcome measure being reduction in antibiotic prescriptions (5).

## Methods

### Clinical trial procedures

The trial methodology is described elsewhere (5). Briefly, PHCs in three Operational Districts (ODs) were randomized 1:1 to one of two arms: 15 PHCs in the intervention arm (EDAM-guided management with associated training) and 15 PHCs in the control arm (routine care). Each PHC aimed to recruit 152 patients, totaling 4,560 participants aged ≥1 year. Prior to the trial, HCWs received two days’ training including clinical scenarios to familiarise them with its use. Enrollment support included access to standard operating procedures (SOPs) via an electronic drive and direct contact with the study team via a Telegram group chat.

### Structured observation procedures

**Figure 1:**
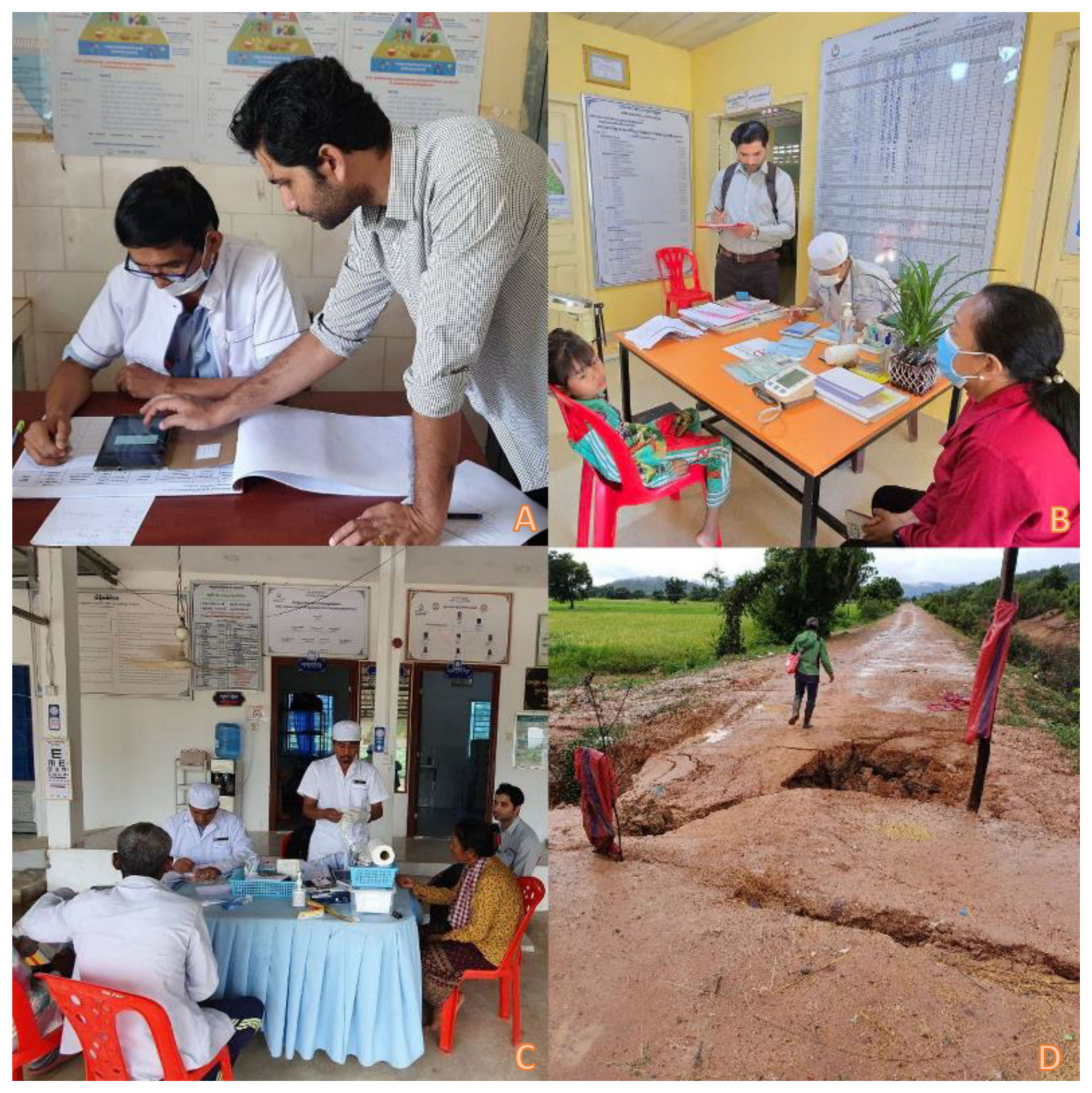
(A) Healthcare workers participating in an on-site refresher training session for the EDAM application. (B) A researcher observes the assessment of a pediatric febrile illness by a healthcare worker. (C) One healthcare worker prepares a malaria rapid diagnostic test (RDT) at the point of care while a colleague concurrently evaluates the other febrile patient. (D) Severe flooding caused by seasonal rain isolates communities, disrupting transportation and complicating health-seeking behaviors. (Photo used with consent from all individuals pictured.)

The mixed-methods study presented here was embedded as part of the trial implementation. In each Operational District, 4-5 PHCs were selected for observation. Selection was non-random, focusing in particular on PHCs where enrolment rates were low and/or where the ratio of screened to enrolled patients was lower than expected. All research staff underwent a two-day standardised training session prior to data collection. This included a detailed review of the EDAM algorithm and study SOPs, hands-on practice sessions (e.g., use of the clicker counter for respiratory rate measurement, blood pressure measurement, pulse oximetry, and CRP testing), as well as training on correct interpretation and error classification. At each PHC, these trained research staff observed 12-15 consultations of enrolled patients, aiming to represent a variety of different years of experience of the HCWs enrolling the patients and different age groups of the enrolled patients. A checklist (Supplementary Appendix 1) was designed to cover several key themes: observation details (including patient socio-demographic data), algorithm utilisation, technical issues, time to form completion, and study procedures. Each individual step of the algorithm was assessed by the research staff (Figure 1, B), with a description of any errors and perceived causes documented for incorrect use. In addition, the time taken to complete each step was recorded. Discrepancies in the clinical information documented in the PHC logbook and the EDAM app were documented and their possible cause evaluated.

### Study outcomes and analysis

The outcome measures were the time taken to complete each section of the algorithm and the overall time to form completion; the frequency, description and possible causes of incorrect use of the app; and inconsistencies in collected clinical data. Outcomes were collated into two key themes according to their implications for future research, namely app-related and contextual challenges.

## Results

### App-related challenges

1. **Time constraints:**
  The median total time to complete the EDAM assessment was 29.1 minutes (IQR 21.0–39.0). Analysis of sub-tasks revealed significant time variation, with the most time-consuming components being danger sign assessment (median 2.0 minutes, IQR 1.3–3.6) and vital sign measurement (median 1.9 minutes, IQR 0.9–5.6). Considerable heterogeneity was observed between PHCs, with total completion times ranging from <11 minutes to >39 minutes (Supplementary Appendix 2). This variability was particularly pronounced on busy days, when some experienced health workers maintained their efficiency, while others appeared to take shortcuts to manage the patient flow. HCWs reported anecdotally that routine febrile illness consultations were typically concluded in under five minutes, highlighting that the algorithmic guidance increased the consultation time over six-fold.
2. **Binary symptom questions:**
  The app used binary questions to assess symptoms and danger signs, which proved challenging for inexperienced HCWs. For example, determining the severity of symptoms such as vomiting or lethargy required a level of experience in clinical judgment that some HCWs lacked. This sometimes led to inaccurate histories and inappropriate referrals, highlighting the need for more nuanced questioning, clearer explanations, and additional training. In response, mid-trial interventions were implemented to address this and other usability bottlenecks identified through observations. Refresher training (Figure-1, A) focused on the assessment of danger signs was initiated at affected facilities. Routine visits after this training suggested a trend toward reduced time spent on the symptom assessment section and fewer observer-documented errors. Similarly, real-time consultation with research staff through Telegram for app navigation coincided with decreased total completion times at several health centres that had previously struggled.
3. **Data quality:**
  Discrepancies between clinical data in the official PHC logbooks and the EDAM app were observed. The most frequent discrepancies involved symptom duration and severity grading. Field observations suggested three primary drivers: (1) Time pressure and parallel documentation burden, leading to shorthand notes in the logbook that were later expanded differently in the app; (2) Recall errors, as health workers sometimes finalised the patient record in the app after the patient had left, relying on memory and (3) Workflow misalignment, where vital signs entered directly into the app were not transcribed into the paper logbook.
4. **Understandability and acceptability of the algorithm:**
  Observer field notes and informal HCW feedback revealed that the underlying structure of the EDAM algorithm was not always intuitive to users. Some HCWs perceived the algorithm as rigid and felt it did not always align with their clinical judgment. These issues were partially mitigated by refresher training and on-site mentoring, underscoring the need for ongoing support to facilitate cognitive acceptance of algorithmic guidance.

### Contextual challenges

1. **Technological barriers:**
  The EDAM app was designed for mobile devices, but its usability depended on familiarity with smartphones. Slower recruitment was observed in PHCs where HCWs lacked experience with smartphone or tablet devices, highlighting the need for digital competency training.
2. **Patient follow-up:**
  Follow-up was complicated by the mobile nature of patients and reliance on family members for communication, particularly among the elderly. Collaboration with village-level HCWs and leaders helped mitigate loss to follow-up, but challenges remained for patients in temporary accommodation and remote areas.
3. **Environmental factors:**
  Extreme weather conditions, such as hot afternoons and flooded roads (Figure 1, D) during the rainy season, affected patient attendance and screening rates, potentially introducing selection bias. These factors highlight the need for flexible trial designs that account for adverse environmental conditions.
4. **Staff shortages:**
  PHCs often faced staff shortages, with HCWs who had received training on the app diverted to administrative tasks or community outreach programs. This limited consistent use of the app and highlighted the need for additional staffing, training and task-sharing strategies.

### Lessons learned

1. **User-centred design:**
  The trial underscored the importance of designing digital health support tools that align with HCW workflows and capabilities. Pre-trial training and iterative feedback were critical to improving usability and acceptability.
2. **Stakeholder engagement:**
  Regular contact with the PHD and inclusion of PHD officials in field visits motivated HCWs and accelerated patient enrollment. Collaboration with a local NGO (Action for Health Development, AHEAD), and village leaders also facilitated follow-up and community engagement.
3. **Balancing efficiency and comprehensiveness:**
  The time-intensive nature of the app posed a significant acceptability barrier. Future iterations should aim to balance efficiency with the need for comprehensive clinical evaluation, possibly through streamlined algorithms and offline functionality.
4. **Contextual adaptation:**
  The trial highlighted the need for digital health interventions to account for local challenges, such as staff shortages, environmental factors, and technological literacy. Flexible implementation strategies, such as peer-to-peer training and adaptive algorithms may be useful in enhancing the relevance and usability of the app.

## Conclusion

This study yielded useful insights into the challenges and opportunities of implementing a digital health intervention in a rural LMIC setting. Key lessons include the importance of user-centred design, intensive training, stakeholder engagement, and contextual adaptation. While the app showed promise in optimising AFI management, its success depended on addressing time constraints, technological barriers, and local challenges, which may have contributed to the desired primary outcome of reduced antibiotic prescribing not being reached in the trial (5). The findings offer guidance for researchers and policymakers developing and evaluating digital health solutions for low- and semi-skilled HCWs in similar settings. The lessons learnt are valuable for these stakeholders, since digital health interventions intended for rural LMIC contexts are currently uncommon but will likely become more so, and there are unique challenges associated with their implementation and subsequent evaluation.

## Supplementary Material

Supplementery Appendix 2

Supplementry Appendix 1

## Data Availability

All data relevant to the study are included in the article or available upon reasonable request from the corresponding author.
