# Supplementary material for: Lessons from the field: Implementing an electronic clinical decision support app for acute febrile illness in rural Cambodia
Source: Trans R Soc Trop Med Hyg. Author manuscript; Available in PMC 2026 Jun 4. (PMC7619126; doi:10.1093/trstmh/trag035)
Supplement: Supplementry Appendix 1 [file EMS213695-supplement-Supplementry_Appendix_1.docx]

**Appendix 1**

EDAM Observation Checklist

| **Observation details** |  |
| --- | --- |
| Operational District (OD) |  |
| Healthcare centre name |  |
| Date of observation (DD/MM/YYYY) |  |
| Start time of observation |  |
| Age group of observed patient (circle the correct answer) | 1-4 years / 5-14 years / 15+ years |
| **App sections** | |
| Section name | SCREENING |
| Start time |  |
| End time |  |
| Correctly assessed ‘fever in past 24 hours’? | Yes / No |
| Correctly assessed whether patient presented in the past 14 days? | Yes / No |
| Correctly assessed other eligibility criteria? | Yes / No |
| If not correct utilisation: description of error |  |
| If not correct utilisation: possible causes |  |
| Section Name | DEMOGRAPHICS |
| Start Time |  |
| End Time |  |
| Correct utilisation | Yes / No |
| If not correct utilisation: description of error |  |
| If not correct utilisation: possible causes |  |
| Section Name | DANGER SIGNS |
| Start Time |  |
| End Time |  |
| Correct utilisation | Yes / No |
| If not correct utilisation: description of error |  |
| If not correct utilisation: possible causes |  |
| Section Name | VITAL SIGNS |
| Start Time |  |
| End Time |  |
| Correct utilisation | Yes / No |
| If not correct utilisation: description of error |  |
| If not correct utilisation: possible causes |  |
| Section Name | SYMPTOMS |
| Start Time |  |
| End Time |  |
| Correct utilisation | Yes / No |
| Were all relevant symptoms identified and documented? | Yes / No |
| How many symptoms were recorded as being present? | 0 / 1 / 2 / 3 / 4+ |
| If not correct utilization or not all relevant symptoms recorded: description of error |  |
| If not correct utilisation: possible causes |  |
| Section Name | CRP test (if applicable) |
| CRP test indicated? | Yes / No |
| If yes, performed according to SOP? | Yes / No |
| Interpretation of result correct? | Yes / No |
| If not correct utilization or not all relevant symptoms recorded: description of error |  |
| If not correct utilisation: possible causes |  |

| Section Name | VERBAL CONSENT |
| --- | --- |
| Start Time |  |
| End Time |  |
| Correct utilization  (i.e. Was follow-up explained to the patient and verbal consent correctly documented?) | Yes / No |
| If not correct utilisation: description of error |  |
| If not correct utilisation: possible causes |  |
| Section Name | ACTUAL MANAGEMENT |
| Start Time |  |
| End Time |  |
| Did the healthcare worker agree with the suggested management plan? | Yes / No |
| If they did NOT agree with the suggested management plan, did they document this in the EDAM app? | Yes / No |
| Correct utilisation | Yes / No |
| If not correct utilisation: description of error |  |
| If not correct utilisation: possible causes |  |

| **Technical Issues** | |
| --- | --- |
| Device Issues |  |
| App issues |  |
| Connectivity issues |  |
| **Total time to Completion** | |
| Start Time |  |
| End Time |  |
| **Additional information** | |
| Was the diagnosis entered in the EDAM app consistent with the OPD logbook? | Yes / No |
| If not consistent: description of difference |  |
| If not consistent: possible causes |  |
| **Additional comments** | |
|  | |
